# Nutrition and Metabolic Adaptations in Physiological and Complicated Pregnancy: Focus on Obesity and Gestational Diabetes

**DOI:** 10.3389/fendo.2020.611929

**Published:** 2020-11-30

**Authors:** Sara Parrettini, Antonella Caroli, Elisabetta Torlone

**Affiliations:** ^1^S. Maria della Misericordia Hospital, Division of Endocrinology and Metabolism, Perugia, Italy; ^2^Department of Medicine, University of Perugia, Perugia, Italy

**Keywords:** pregnancy, obesity, body composition, gestational diabetes, diet, fetal development

## Abstract

Pregnancy offers a window of opportunity to program the future health of both mothers and offspring. During gestation, women experience a series of physical and metabolic modifications and adaptations, which aim to protect the fetus development and are closely related to both pre-gestational nutritional status and gestational weight gain. Moreover, pre-gestational obesity represents a challenge of treatment, and nowadays there are new evidence as regard its management, especially the adequate weight gain. Recent evidence has highlighted the determinant role of nutritional status and maternal diet on both pregnancy outcomes and long-term risk of chronic diseases, through a transgenerational flow, conceptualized by the Development Origin of Health and Diseases (Dohad) theory. In this review we will analyse the physiological and endocrine adaptation in pregnancy, and the metabolic complications, thus the focal points for nutritional and therapeutic strategies that we must early implement, virtually before conception, to safeguard the health of both mother and progeny. We will summarize the current nutritional recommendations and the use of nutraceuticals in pregnancy, with a focus on the management of pregnancy complicated by obesity and hyperglycemia, assessing the most recent evidence about the effects of ante-natal nutrition on the long-term, on either maternal health or metabolic risk of the offspring.

## Introduction

Pregnancy is a period of physical, hormonal and humoral changes, aimed to ensure the development and the necessary supply of nutrients to the fetus, and to prepare the maternal organism to delivery and breastfeeding. However, in the woman’s life, this biological phenomenon is considered as a window on the future health of both mother and offspring (1).

It is clear that maternal metabolic alterations, such as obesity and diabetes, have negative consequences on the offspring, for either embryonic teratogenic effect or outcomes at birth, such as mortality or pre-term birth. Moreover, there is an increasing interest in understanding the effects on long-term susceptibility of chronic-degenerative diseases.

In the 1980s, Dr. D. Barker and collaborators from the University of Southampton (UK), recovering the historical birth data in Hertfordshire (UK), introduced the pioneering concept that the origin of disease in adulthood could be strongly associated with exposure to an adverse uterine environment during pregnancy, a low birth weight and a related increase in the risk of offspring morbidity and cardiovascular mortality (2, 3). This phenomenon, better known as “Barker hypothesis”, identifies a development plasticity; therefore, adverse conditions such as maternal malnutrition in early organogenesis can permanently change the structure of organs and systems, as stated by “fetal programming”.

According to a “thrifty genotype”, Barker’s hypothesis reflects a discrepancy between a uterine restriction environment and a postnatal abundance condition in the pathogenesis of obesity (4). The fetus responds to a “poor” environment with irreversible changes in its development trajectory and growth restriction (5); afterwards, in childhood or later, the organism loses the ability to adapt itself to a richer environment, and creates the basis for the development of diseases in adulthood (6).

More than 20 years earlier, N. Freikel had already proposed the key role of the uterine environment in influencing development, and hypothesized that *in utero* over-nutrition was decisive in the long-term.

Discussing within the context of diabetes in pregnancy, he introduced the concept of fuel-mediated teratogenesis, i.e. alterations during cell differentiation, and fetal organogenesis induced by excessive exposure to nutrients of the fetal-placental unit, which have immediate, but mostly long-term consequences on metabolic and anthropometric functions (7).

In recent years, this topic extended to the theory of “developmental origins of health and disease” (DOHaD). This definition underlines the role of both pre- and post-natal environments in shaping developmental trajectories on long-term health (8).

To date, consistent data confirm the pivotal role of maternal nutritional status and diet for the fetal epi-genotype and the resulting phenotype (9–12). Obviously, early exposure (which confers an increased risk of obesity, diabetes, and other metabolic diseases in adult life) unavoidably cause persistent changes in metabolism and neuro-endocrine functions, which leads to this susceptibility in adulthood.

Intriguingly, the relationship between nutrient intake and future health is not limited to the maternal diet during gestation, but it should be framed within a transgenerational heritage that marks the future risk of progeny. A fetus does not depend only on the mother’s diet during pregnancy, as it would be a dangerous strategy for its survival. Rather, it thrives on stored nutrients and on turnover of proteins and fats in maternal tissues, which are obviously related to maternal body composition and reflect her nutrition throughout life. A woman owns all her oocytes since she was born, therefore even before conception the quality of the female gametes will reflect the nutritional status of her mother. The critical 1000 days of development, which will determine the health for the rest of life, reflect 100 years of “nutritional flow” (13).

Specific maternal conditions during the pre- and peri-conceptional period (mostly obesity and excessive weight gain during pregnancy) are associated with Large-for-gestational-age (LGA) infants, obesity, and impaired glucose metabolism in children and subsequently with increased cardiometabolic risk in adults. Nutritional status during pregnancy cannot disregard the assessment of pre-pregnancy nutritional status.

This review will address the fascinating process that, starting from the functionality and anatomy of the placental-fetal unit, let to ensure the full-term delivery of a healthy newborn. Moreover, we will review the nutritional recommendations and the use of nutraceuticals in pregnancy, with a focus on the management of pregnancy complicated by obesity and hyperglycemia. Finally, we will focus on the most recent evidence about the effects of ante-natal nutrition on the long-term, on either maternal health or metabolic risk of the offspring.

## Placenta: A Metabolic Organ

The placenta is comprised of specialized epithelial cell types, collectively referred as trophoblast cells, situated among mesenchymal cells and vasculature, at the maternal-fetal interface. It is a kind of metabolic mirror of the health status of both fetus and mother, responding to the environment in order to maintain the fetal viability (14). The placental barrier is formed by two layers that regulate the transfer of nutrients from maternal to fetal circulation: the syncytiotrophoblast (SCTB), which line the villi and constitute the transporting epithelium of the placenta, with two polarized membranes (the microvillous membrane—MVM—facing maternal circulation, and the basal plasma membrane—BM—on the fetal side), and the fetal capillary endothelium (15–17).

Glucose is the primary energy substrate for fetal-placental unit; in fact, in the absence of appreciable gluconeogenesis, placental glucose transport constitutes the only supply for the fetus.

Three types of protein solute carriers are involved in glucose transport: the facilitated diffusion transporters (GLUTs, SLC2), the sodium-coupled symporters (SGLT, SLC5) and the more recently identified SCL50 transporters (18). GLUT1 is the primary glucose transporter, with a 3-fold greater density in MVM, increasing throughout gestation to meet the higher need for fetal growth (19). Placental-to-fetal glucose transfer (PGT) capacity increases about 8-fold over the pregnancy (20), thus placental glucose uptake increases with advancing gestation (21).

The hyperglycemic environment of a pregnancy complicated by hyperglycemia in pregnancy (HIP) alters the development of the placenta and several morphological alterations have been described, such as maternal vascular malperfusion, fetal thrombosis, a vasoactive molecules imbalance, and boosted oxidative stress (22–24). Placentas from mothers with HIP are frequently larger, with an increased placental weight and placental-weight to birth-weight ratio irrespective of glycemic control (25). Epigenetic studies of Genome-wide methylation analysis highlighted that metabolic disturbances associated to Gestational Diabetes (GDM) may also affect DNA methylation and gene expression profile, particularly at arterial (AEC) and venous endothelial cells (VEC) level, with respect to cell morphology, cellular movement, and those inducing actin organisation and barrier functions (26).

Glucose transport capacity seems to be increased in GDM placentas, but with conflicting results about GLUT1 expression and activity in MVM and BM (24). At the same time, it has been demonstrated that women with Type 1 Diabetes (T1D) and first trimester hyperglycemia, showed higher expression of GLUT1 in the BM compared to healthy pregnancies (27).

Aminoacid uptake across cellular membranes is mediated by active transporter processes (enzymatic mechanism mediated by ATPase), and can be categorized into distinct systems. For each system, an accumulative transporter increases intracellular aminoacid concentrations by uptake against the concentration gradient, co-transporting extracellular sodium. Instead, some exchangers switch amino acids between the intracellular and extracellular compartments across the BM (28). Fetal aminoacid concentration are generally higher than in maternal compartment, reflecting this active transport.

At least 15 different amino acid transporters were identified, but System A transporter is the most involved (29). The System A amino acid transporter facilitates uptake of small non-essential neutral amino acids such as alanine, glycine, and serine against their concentration gradient. Cytokines and hormones regulate placental System A activity, such as insulin, leptin, interleukin (IL)-6, and Tumor Necrosis Factor (TNF)-α, stimulating activity of the transporter (17). It appears that there is a direct link between down-regulation of placental amino acid transporters and restricted fetal growth in IUGR (29). System L (LAT) is a sodium-independent exchanger for large neutral amino acid transport, which exchanges non-essential amino acids for essential ones with branched or bulky chains, such as leucine, and it is stimulated by glucose and insulin (16). Thus, the combined activities of Systems A and L are critical in the fetal supply of neutral and essential amino acids for fetal biosynthesis and metabolism.

The sodium-dependent System β (TAUT) plays a key role in the transplacental transport of taurine to the fetus, where it is essential for antioxidant processes and neurological development (30). Moreover, the high affinity Excitatory Amino Acid Transporters (EAATs; System X_AG-_) exchange glutamate across the MVM and BM, being converted to glutamine in the placenta (31): abnormal glutamine and glutamate transporter activity is recently showed as part of placental dysfunctions in IUGR (32).

Other transporters for cationic and anionic amino acids have been detected in the human placenta, although their specific role in determining the fetal growth is less well studied (33)

Finally, a number of mechanisms ensure adequate transport of free fatty acids (FFA) to the fetus. Fatty acids are a source of energy, constitute an essential structural element of cellular membranes, and are important for the development of specific tissues (17). The fetal source of fatty acids is either triglyceride (TG)-rich maternal lipoproteins, such as chylomicrons and VLDL, or FFA bound to albumin. Albumin–FFA complexes interact with fatty acid binding proteins (FABP) in the microvillous plasma membrane (MVM), uptaking FFA into the syncytiotrophoblast cells. TG in maternal lipoproteins, in particular Very-Low-Density Lipoproteins (VLDL), are hydrolysed into FFA by lipoprotein lipase (LPL) expressed in the MVM. FFA is subsequently transferred across the MVM. Alternatively, maternal lipoproteins interact with LDL/VLDL receptors in MVM resulting in endocytosis and intracellular hydrolysis, which releases FFA. Intracellularly, FFA are transported bound to FABPs (34). Maternal lipid metabolism changes during pregnancy, placental LPL activity increases, and plasma FFA concentrations rise rapidly during the third trimester thus representing the main class of lipids crossing the placenta (35).

In diabetic pregnancy, a wide range of disturbances in lipid metabolism have been described and maternal lipids seem to be the strongest determinants of fetal growth in GDM newborns (36). Both maternal triacylglycerols (TAGs) and non-esterified fatty acids (NEFAs) are positively correlated with neonatal weight and fat mass (FM) in GDM pregnancy, indicating that maternal dyslipidemia in GDM may enhances the availability of lipids to the fetus (36, 37).

Maternal diet and metabolic status alter placental lipid transport and biology (38). First, dietary composition may directly alter the pool of fatty acids available for uptake by the placenta; however, dietary fats may alter placental lipid transfer indirectly, as omega-3 fatty acids which is believed to modulate placental oxidant stress and inflammatory status, then lipid transport (39). Maternal GDM and obesity have been reported to alter placental fatty acid transfer, although further progress will be need in understanding the mechanisms of specific placental alterations on lipid transport and metabolism (38).

Recent evidence did not support an increased maternal-to-fetal transfer of glucose and fatty acids in complicated pregnancies in late weeks (40, 41). The placental changes throughout pregnancy may reflect adaptive responses to protect the fetus from the adverse metabolic environment, such as fatty acids storage in lipid droplets by the syncytiotrophoblast; however, when more marked metabolic conditions are determined, such as grade III obesity or severe maternal hypetriglyceridemia, the placenta is no longer able to accumulate lipids, with a consequent spillover towards the fetus (42).

**Figure 1:**
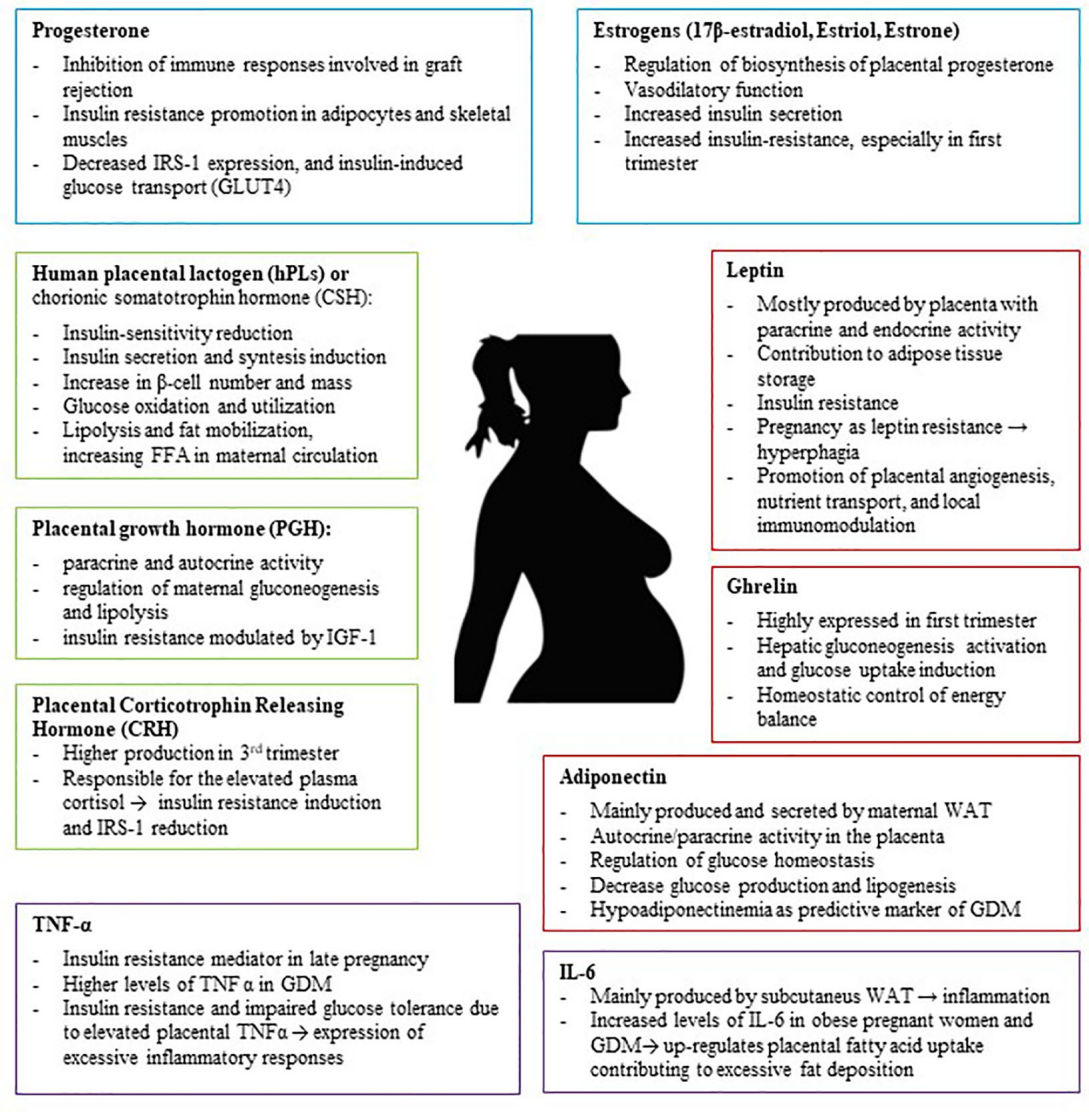
The Placenta is an endocrine organ which produces several metabolic proteins (Leptin, Adiponectin, Ghrelin), peptide hormones (hPL, PGH, CRH), steroid hormones (Progesterone, Estrogens), and cytokines (TNFα, IL-6) which have significant influence on establishing and maintaining a healthy pregnancy, regulating glucose and lipid metabolism and their adaptation throughout pregnancy. IRS-1, insulin receptor substrate 1; GDM, Gestational Diabetes; TNF-α, Tumor Necrosis Factor α; WAT, white adipose tissue.

Eventually, the role of the placenta is not simply to mediate solute transfer: it is a central metabolic and endocrine organ of pregnancy. Thus, this organ produces numerous hormones which have significant influence on establishing and maintaining a healthy pregnancy, regulating glucose and lipid metabolism and their adaptation throughout pregnancy (43) (**Figure 1**). In pregnancies complicated by obesity or GDM, the expression levels of placental molecules are disrupted, and this lack of balance may be associated with other metabolic complications (44).

## Metabolic Adaptations in Pregnancy

Pregnancy has a diabetogenic effect on metabolism. Thus, since mid-pregnancy, placental-derived hormones reprogram maternal physiology to achieve an insulin resistant state, reducing insulin sensitivity (10). Maternal insulin resistance (IR) (mostly expressed at skeletal muscle and adipose tissue level) is physiological in pregnancy and is critically important to maintain the maternal fuel supply to support the growing fetus, mostly during the third trimester.

Pregnancy is characterized by a complex endocrine-metabolic rearrangement. The first phase of gestation, defined as “maternal anabolic phase”, is characterized by an increase in maternal energy reserves, mainly in the form of lipids, storing the substrates to meet the maternal-fetal needs of advanced gestation and breastfeeding. The second phase of pregnancy is defined as “maternal catabolic phase” or “fetal anabolic phase”, because it is aimed at fetal growth, with a significant reduction of insulin sensitivity and an increase in maternal concentrations of glucose and FFAs (45, 46).

Catalano and co-workers demonstrated that the metabolic dysfunction includes impaired insulin response in peripheral tissues, decreased hepatic suppression of glucose production during insulin infusion, and decreased insulin stimulated uptake in skeletal muscle (45). In physiological pregnancy, insulin-mediated whole-body glucose disposal decreases by 50%, mediated by a decrease in insulin tyrosine-kinase receptor phosphorylation and tyrosine-kinase receptor activity, and a impaired ability to translocate the GLUT4 transporter to the surface of muscle cells (47).

During pregnancy, a progressive reduction in blood glucose develops. A fasting condition leads to a further progressive reduction in fasting blood glucose, which is more marked in the third trimester (from 75 to 65 mg/dl) (48). Despite the reduction in blood glucose, and a 3.0- to 3.5-fold increase in fasting insulin, maternal hepatic production of glucose (mainly gluconeogenesis) is increased. In fact, in hyperinsulinemic euglycemic clamp studies with an insulin infusion rate of 1.0 mU/kg/min, there is a significant 30% increase in basal endogenous glucose production at 34 to 36 weeks’ gestation in comparison with 12–14 weeks’ gestation measurements in women with either normal glucose tolerance or GDM, without differences between the groups (49, 50). The increase in basal endogenous glucose production (gluconeogenesis and glycogenolysis) is a necessary adaptation of maternal metabolism to meet the increasing carbohydrate requirements of the fetus and placenta. However, women with obesity or a history of GDM enter pregnancy with pre-existing IR that worsens with advancing gestation; overweight/obese women have decreased insulin sensitivity as compared with lean women, although both groups have a similar 50% decrease of insulin sensitivity during gestation. Catalano and colleagues demonstrated with the euglycemic hyperinsulinemic clamp, that in GDM women there was only an 80% suppression of hepatic glucose production, as compared to healthy pregnant women, who had a 95% suppression of glucose production (51, 52). As a consequence of insulin resistance, fat oxidation increased 220% in both lean and obese women (53).

With respect to maternal lipid metabolism, during the anabolic phase of first and second trimesters, the increased estrogens, progesterone and insulin concentrations promote lipid deposition: FA synthesis and lipoprotein lipase expression increases, which facilitated cellular uptake of circulating triacylglycerols (TAGs). FAs, TAGs, cholesterol, and phospholipids blood levels gradually increase and this continues through the third trimester (43). In the third trimester a metabolic shift though a catabolic state occurs and the further deposition of fat mass stops: LPL activity, lipolysis from adipose tissue (especially in fasting) and FFA levels increases, lipids become the major energy source, while glucose and aminoacids are retained for the fetus (54).

In fasting status, there is a rapid diversion of maternal metabolism towards lipid oxidation, with both an exaggerated increase in FFA and the production of ketone bodies. The reduction in blood glucose, insulinemia and the increase in the plasma concentration of FFA and β-hydroxybutyrate occur earlier in pregnant than in non-pregnant women. The increased lipolysis and ketogenesis use stored fats as energy. All these modifications of the intermediate metabolism aim to save glucose for the fetus.

From a clinical point of view, the most important problem of this “accelerated starvation” is the increased concentration of circulating ketones, freely crossing the placenta and making themselves available for hepatic and fetal cerebral oxidation. This unrestricted arrival of ketone bodies from maternal to fetal circulation is an essential adaptation, which guarantees embryonic brain development under condition of nutrient deficiency. However, this adaptation could also have negative effects on fetal development, since maternal hyperketonemia has been associated with increased incidence of fetal malformations, impaired neurophysiologic development, as well as still birth (46, 55). For this reason, it is important to prevent fasting ketosis during pregnancy (56).

### Placental Role in Metabolic Adaptations

The placenta plays a pivotal role in the development of insulin resistance in pregnancy. Among the placental hormones involved in this complex metabolic reorganization, the human placental lactogenic hormone (hPLs) plays a decisive role in the regulation of maternal glucose metabolism.

HPL is secreted by the placenta and introduced only into the maternal circulation since the sixth week of gestation, to reach higher blood levels in the second half of pregnancy. HPL is a peptide hormone, belonging to the same family of the growth hormone (GH) and prolactin, and, as the latter, it exerts a lipolytic action and diabetogenic effects: the consequent increase of FFA are functional to the mother’s greater energy needs and contribute to insulin resistance. Furthermore, hPL seems to have a direct plastic action on the β-cell mass, and induce insulin secretion (43, 57). Pregnancies affected by metabolic conditions, including obesity and diabetes, are related to alterations in hPL secretion: obesity is often associated with lower PL serum concentrations, whereas GDM results in increased hPL blood levels. Moreover, disruptions in hPL secretion are thought to be associated with an increased prevalence of gestational complications, such as placental dysfunction, and abnormalities in fetal growth (positive correlation with birth weight) (58).

The variant of the placental growth hormone (PGH), mostly expressed and secreted from placental syncytiotrophoblast, contributes to insulin resistance replacing pituitary GH in maternal circulation, and acts by facilitating the mobilization of maternal reserves, such as lipolysis and gluconeogenesis, for fetal growth (59). Moreover, it mediates insulin resistance by direct modulation of Insulin Like Growth Factor 1 (IGF-1) (60). Diabetic pregnant women did not show correlation between the changes in PGH levels and insulin levels (61).

Both White Adipose Tissue (WAT) and placenta act as endocrine organs in pregnant women, secreting adipokines and cytokines such as leptin, adiponectin and Tumour Necrosis Factor α (TNF-α) that contribute to metabolic reprogramming (62, 63).

Circulating levels of leptin gradually increase throughout gestation. Ladyman et al. demonstrated a resistance to anorexigenic effects of leptin during pregnancy (64). Leptin resistance is important to maintain increased energy intake to support fetal growth in the second and third trimester (65). This also contributes to adipose tissue storage in early and mid-pregnancy to prepare for lipid mobilization during the catabolic phase of late pregnancy. Leptin is usually increased in obese pregnant women and in those with GDM, then early hyperleptinemia is a predictor of GDM risk (66). Moreover, the hyperinsulinemia in GDM could further stimulate the production of LPT which in turn would amplify the inflammation (67). This action creates a vicious circle that perpetuates the inflammatory state and intensifies insulin resistance (63).

Adiponectin is almost exclusively synthesized by adipocytes, but it is expressed also in syncytiotrophoblast (68), exerting a potent insulin sensitising effect. Macrosomia and LGA new-borns are likely to be associated with reduced adiponectin levels, in fact maternal adiponectin showed an inverse association with neonatal birth weight (69). Moreover, adiponectin concentration decreases in GDM: normal-weight women with GDM showed lower adiponectin levels, and hypoadiponectinemia could be a predictive marker of GDM development in early gestation (70). Women with adiponectin concentrations below the 25th percentile in the first trimester were more likely to be diagnosed GDM (45).

Likewise, TNF-α is a proinflammatory cytokines also produced by the placenta, apart from skeletal muscle, aiming to exacerbate insulin resistance; it seems to play a significant role in the development of insulin resistance (52), with higher expression of TNF-α receptors in placenta of GDM women (71).

Placenta produces several other adipokines (RBP-4, Resistin, Chemerin, Apelin, Omentin, FABP-4) involved in the modulation of the metabolic processes and insulin resistance in pregnancy, however their role on the pathogenesis of GDM is lacking (63, 72).

All metabolic adaptations in pregnancy are crucial for the proper fetal development. Thus, several biomolecules including glucose, fatty acids, ketone bodies, hormones and adipokines maintain a proper balance, in which the placenta plays a central role.

## Body Composition and Gestational Weight Gain

Pregnancy is an extremely dynamic phenomenon, related to the progressive addition of proteins, fats, water and minerals in the constitution of both fetal and maternal structures. The product of conception (placenta, fetus, amniotic fluid) accounts for about 35% of gestational weight gain (GWG) (73). Epidemiological studies have shown an association between GWG and the risk for adverse pregnancy outcomes including preterm birth, impaired fetal growth, infant death (74, 75).

Weight gain is the major determinant of the increase in energy demands during pregnancy. The negative effects of both excessive and inadequate GWG on maternal–fetal outcomes have been taken into account by the Institute of Medicine (IOM) that developed universal guidelines for healthy GWG on the basis of pre-gravid body mass index (BMI) (76). However, today there are still many disputes, even regarding current indications.

In fact, maternal body composition changes over the trimesters to support fetal growth. In the first months of gestation, the changes in maternal body composition reflect preparation of the maternal body for fetal development. Specifically, the uterus and breast tissue of the maternal unit grows and blood volume expands. In late pregnancy, a more pronounced growth of the fetal unit (e.g., fetus, amniotic fluid, and placenta) occurs along with maternal tissue and further blood volume expansion. Moreover, a large and variable change in Fat Mass (FM) and Fat Free Mass (FFM) was observed in late gestation (73, 75, 76).

The deep metabolic changes that occur during pregnancy (an anabolic state in the first two trimesters, and a maternal catabolism in the third), linked to hormonal variations, contribute to progressively increase energy demand. In the second half of gestation, the rapid fetal growth and the higher cardio-vascular and respiratory work, increase basal metabolic rate (BMR) by 60%.

The energy demand in pregnancy is determined by 3 components (54, 76):

- Energy deposited for the new synthesis of tissues (placenta, uterus, amniotic fluid, fetal blood): it is really a deposition of protein mass, estimated at about 597 g (for a woman with an optimal weight gain of 12 kg).
- Energy deposited as maternal fat mass (mainly between 20–30th week).
- Energy required for the maintenance and growth of the newly formed tissue, related to the increase in basal metabolic rate (BMR).

Maternal FM is the most variable component of GWG, which mostly contributes to the energy costs of pregnancy and positively correlate with GWG (77). Lean and fat mass significantly increase in both lean and obese women, but the increase in FM is greater than FFM in lean women, although there is a significant 23% increase in resting energy expenditure (REE), without significant difference between groups (53).

A study of Berggen and colleagues demonstrated that in obese women GWG correlated strongly with FM change (p<0.001), thus, excessive GWG with respect to IOM recommendations was primarily associated with maternal fat, but not lean body mass accrual (78). Another Canadian study showed in 1820 pregnant women that excessive GWG was a significant risk factor for higher FM accretion during pregnancy, but also for higher 3-months postpartum fat retention, irrespective of pre-pregnancy BMI (79). These results might explain why excess GWG, or fat mass accrual, is associated with long-term obesity and metabolic dysfunction.

Although a total additional energy demand of ~ 76,000 kcal has been estimated during the whole pregnancy (correlated to a maternal weight gain of about 12 kg), there is an inter-individual variability of energy expenditure, linked not only to GWG but also to the pre-nutritional state, clearly expression of the plasticity of the metabolic adaptations to the actual requirements. For instance, women from developing Countries, who have an inadequate pre-pregnancy nutritional status and low fat reserves, adapt to pregnancy by reducing their BMR, in order to use their energy for fetal needs. In those starting in a good state of nutrition and of normal weight, the BMR progressively increases over the course of the trimesters, as already described (80, 81). Overweight women increase BMR over 30% (82).

The amount and composition of appropriate GWG significantly differs between women with different pre-pregnancy BMI. Current weight gain guidelines have not been expanded to include recommended gains in FM and FFM.

Body composition could be commonly assessed with methods including anthropometry, densitometry (air displacement plethysmography, underwater weighing), and hydrometry (isotope dilution, bioimpedance analysis), but when these techniques are applied to pregnancy, they require specific adjustments to their assumptions, mostly for the hydration of FFM which is flexible and progressive throughout gestation for tissue fluid imbibition (75, 83). Understanding the maternal body composition changes, may contribute to predict pregnancy-related complications; furthermore, the correlation between maternal body composition and energy balance will let to develop adequate energy intake recommendations, aiming to facilitate an appropriate weight gain throughout pregnancy.

## Obesity in Pregnancy: From Physiopathology to Metabolic Derangements

Maternal obesity is a metabolic condition that is increasingly becoming a public health problem, affecting a large number of women with consequences for the health and well-being of both mother and child: up to 30% of women in reproductive age are obese (84) and in recent years the prevalence of women starting pregnancy with a BMI ≥ 30 Kg/m^2^ has steadily increased (85).

Pregnancies complicated by maternal obesity are associated with adverse outcomes, including increased risk of GDM, pre-eclampsia, preterm birth, need of cesarean section, infections, and post-partum haemorrhage. Furthermore, maternal obesity seems to play a key role in “planning” the development of metabolic diseases in adulthood (53, 86) (**Table 1**).

**Table 1:** Maternal obesity: consequences and short- and long-term health risks for both mother and child.

	Mother	Child
Pre-conception	- Low fertility - Early pregnancy loss	
Pregnancy and delivery	- GDM - Pre-eclampsia, Eclampsia - Operative delivery - Cesarean delivery - Pre-term birth	- Fetal Distress Syndrome - Macrosomia, LGA newborn. Increased adiposity. - Shoulder dystocia e perinatal injuries - Stillbirth
Post-partum	- Post-partum depression - Deep vein thrombosis (DVTs). - Hypertension - Dyslipidemia	- Reduced breastfeeding - Increased adiposity
Long-term effects	- T2D - OSAS	- Obesity, diabetes, dyslipidemia, hypertension: Metabolic Syndrome (MetS) - Cardio-vascular diseases - Cognitives disorders

In fact, newborns of obese women have an increased risk of overgrowth, and maternal obesity accounts for a greater number of LGA infants than pregnancies complicated by GDM (53, 87). It has been demonstrated that infants of GDM and obese women weigh more at birth than normal glucose tolerant or normal-weight women, respectively; in both cases, it depends on an increase in fat mass and not lean body mass (87, 88).

Maternal BMI has been proved the main predictor of neonatal adiposity. The Healthy Start Study showed a positive and independent associations of continuous maternal pre-pregnancy BMI and GWG with neonatal adiposity measures, with period-specific rates of GWG in early-, mid-, and late pregnancy independently associated with neonatal FM and the percentage of body fat (89).

A sub-analysis of HAPO study demonstrated that maternal obesity was a strong independent predictor with adverse perinatal outcomes (newborn weight and body fat ≥90th percentile, preeclampsia). The authors found a higher risk of preeclampsia in obese non-GDM (OR 3.91) than in normal-weight GDM women (OR 1.74). However, obesity in addition to GDM was associated with a greater risk of preeclampsia than either factor alone (OR 5.98). These results showed that maternal obesity and GDM alone and in combination are independently associated with adverse pregnancy outcomes, with more than an additive effect, implicating other potential mechanisms such as inflammation (90).

A model of fetal overgrowth in obese women may consider obesity as a “fat sickness”, with a low-grade subclinical systemic and tissue-specific inflammation causally linked to insulin resistance (91). When pre-pregnancy insulin resistance in obese women superimposes to the pregnancy-related insulin resistance, it leads to an increased insulin response, which affects early placental growth and gene expression. The release of placental cytokines and hormones (i.e. hPL) are enhanced, with a crosstalk decreasing insulin sensitivity in maternal tissue and a consequent increase in nutrient availability, which contributes to the fetal growth and adiposity (53).

At the same time, the placenta is thought to contribute to excessive neonatal fat already in early pregnancy. Placental volume at the end of the first trimester is associated with neonatal birth weight; besides, placentas of neonates born LGA have a greater volume than appropriate-for-gestational-age (AGA) ones (92). It has been proposed a role of early hyperinsulinemia, as well as hyperglycemia in the first trimester, in placental growth, through insulin receptors activation at the syncytiotrophoblast surface. In fact, higher placental volume and surface area may define the total number of glucose transporters and enhance transplacental nutrient flux, which in turn is associated with fetal and neonatal phenotype and excessive growth (42). Moreover, the transcriptome pattern of trophoblast cells from placenta of obese women is deeply modified compared with lean ones. Obesity associated with hyperinsulinemia and insulin resistance impairs the global gene profiling of first trimester placenta, particularly several sets of genes which regulate cell cycle parameter, lipid metabolism and mitochondrial activity, leading to a sort of mitochondrial disfunction at term (93).

**Figure 2:**
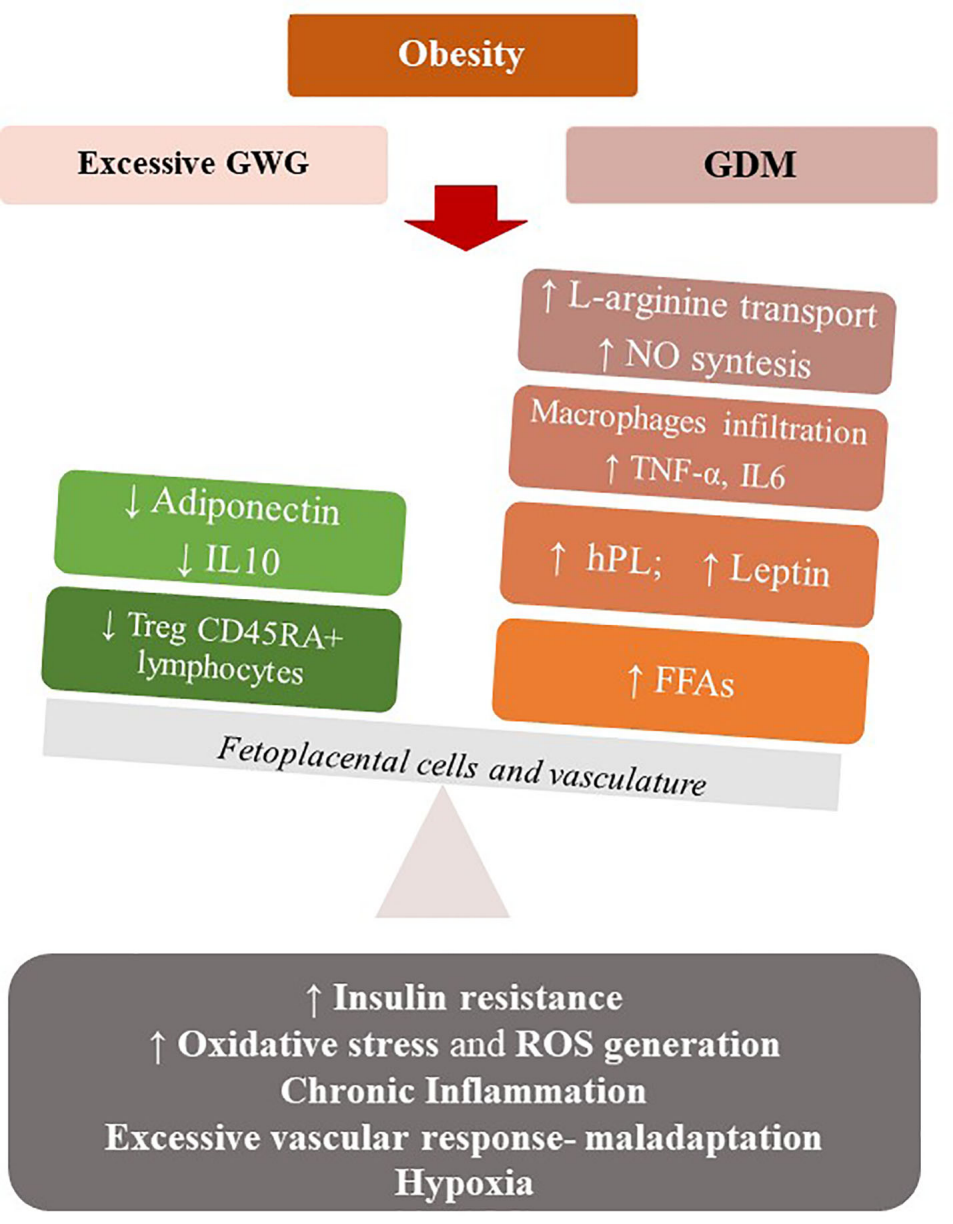
Maternal obesity, excessive GWG and GDM may be associated with a state of low-grade chronic inflammation that configure a metabolic condition (Gestational Diabesity) sharing a common pathogenetic substrate: the loss of balance between the physiological pro- and anti-inflammatory responses of pregnancy and an enhanced pro-inflammatory response. Increased levels of leptin and TNF- α, a state of hypoxia, abnormal oxidative stress, vascular maladaptation and enhanced ROS generation result in insulin resistance in the feto-placental vasculature, with reduced levels of adiponectin and IL-10 and increasing NO bioavailability. At the systemic level, there is a de-regulation of the immune system, with reduction of Treg cells and their tolerogentic activity of immune-suppression. hPL, Human Placental Lactogen; TNFα, Tumor Necrosis Factor α; FFA, Free Fatty Acids; IL-6, Interleukin 6; IL-10-Interleukin-10; ROS, Reactive oxygen species.

Maternal obesity shares with excessive weight gain a combination of an exacerbated pro-inflammatory state and a feto-placental endothelial dysfunction (nitric oxide and endothelin-1 imbalance), that may expose mothers to develop GDM. Maternal obesity, excessive GWG and GDM may be associated with a state of “meta-inflammation” (94) that configure a new metabolic condition recently referred to “Gestational Diabesity” (95, 96) (**Figure 2**).

Particularly, in normal pregnancies the equilibrium between the pro- and anti-inflammatory responses of the feto-placental cells is needed. In Gestational Diabesity, the pro- and anti-inflammatory responses loss their equilibrium and the pro-inflammatory response predominates: increased levels of leptin and TNF-α, a state of hypoxia, abnormal oxidative stress, and enhanced ROS generation result in insulin resistance in the feto-placental vasculature, while the reduced levels of adiponectin and IL-10 and increasing NO bioavailability are demonstrated in this tissue (93, 96). This chronic low-grade inflammatory state, the increased insulin resistance and a vascular maladaptation as excessive vascular response to vasoconstrictors, in a context of early weight gain and high-calorie diet, can become the common pathogenetic substrate of diseases such as GDM and preeclampsia (97). Similar to what observed in the non-pregnant population, excessive GWG, GDM and preeclampsia in pregnancy represent a pattern of conditions that mimic the Metabolic Syndrome. Thus, a Metabolic Syndrome of pregnancy (PregMS) has been proposed as condition including an increased risk of hypertensive complications, metabolic disturbances of nutrient metabolism, and inflammation, associated with negative feto-maternal outcomes (97).

Although these pregnancy-related conditions (PregMS) are likely to clinically resolve after delivery, these women may still maintain a subclinical metabolic disorder and are at increased risk of Metabolic Syndrome in later life, particularly if there is increased post-partum weight gain. Women with excess GWG retain the most weight postpartum because of the higher FM accretion (79), and mothers with elevated preconception BMI are more likely to have postpartum weight retention (98, 99): only about 11% of obese pregnant women return to their preconception weight within 5 years postpartum (100). Furthermore, the postpartum period becomes the pre-conceptional period of next pregnancy: a higher GWG exposes the mother to begin next pregnancy with a higher BMI, placing herself at higher risk for excessive GWG, in a vicious cycle leading to increased prevalence of overweight and obesity in reproductive and then postmenopausal age women (99).

Therefore, the nutritional management of GWG represents the main strategy to limit the metabolic trajectory towards a reduction of both maternal and neonatal risk factors (central adiposity, dyslipidemia, glucose intolerance and, in the future, obesity, T2D, and metabolic syndrome).

## Nutrition in Pregnancy: A Challenge in Obese Women

The achievement of an adequate GWG and the satisfaction of nutritional requirements are the goals of proper nutrition in pregnancy complicated by obesity. However, specific nutritional recommendations for obese pregnant women are lacking, and there is controversy about the correct GWG in women with BMI ≥ 30 Kg/m^2^.

### Gestational Weight Gain in Obesity

Most et al. showed that GWG in pregnancy differs by obesity classes and trimesters (101). Based on previous data of body weight and composition for pregnant women up to class 1 obesity, a linear model to predict the changes in body composition (GWG, FFM, and FM) for women with obesity class 2 and 3 and with an appropriate total GWG were elaborated. Assuming a FFM accretion comparable to the other BMI classes and the inverse association between pre-gravidic BMI and FM gain throughout all BMI classes, their model suggested that maternal FM should be lost during pregnancy in cases of morbid obesity (BMI ≥ 40 kg/m^2^) in order to optimize health outcomes (75). Body composition studies with air plethysmography confirmed that in obese women with non-excessive GWG (according to IOM), body weight was affected by changes in FM, and an inverse association between pre-gravidic BMI and FM gain was observed, with women with class 3 obesity gaining less, or loosing body weight and FM, with respect to class 1 or 2 (101).

Studies on energy expenditure highlighted with rigorous methods how the recommended rate of weight gain in obese pregnancies was accomplished when the daily energy intake was maintained from early pregnancy throughout the second and third trimesters and did not exceed total energy expenditure (102). In fact, in pregnant women with obesity the energy requirement for fetal development should be compensated by mobilization of maternal FM, with no adverse effects in maternal or fetal outcomes: therefore, they should not consume additional energy. The authors proposed a daily calorie deficit of about 100 kcal for the recommended weight gain (102). This recent evidence implies that the increase in energy requirements in the second and third trimester, as recommended by both International and Italian guidelines (103, 104) could not apply universally to all BMI classes.

Moreover, FM accumulation was higher in the second as compared to the third trimester and was significantly different among obesity classes (P = 0.002): this confirms that efficient prevention strategies and nutritional management should be initiated early in pregnancy (75).

Recently, on the basis of these studies (101, 102) and of evidence on the relationship between GWG and optimal neonatal birthweight centiles (105), a revision of the recommendations on weight gain for the BMI classes of obesity has been proposed. Specifically, an increase of 2.5–7 kg in women with class 1 obesity, ≤ 4.5 kg for pregnant women with class 2 obesity, and the maintenance of pre-gravidic weight if BMI ≥ 40 Kg/m^2^ (Class 3 obesity) were hypothesized. Obviously, to confirm this view, further studies will be needed, also aimed at assessing the role of diet quality and dietary patterns.

Other studies concerning the effect of maternal weight gain on obstetric and neonatal outcomes support this argument. Bodnar et al. found that GWG between 2.2 and 5 kg in women with severe obesity (Class 3) was associated in less than 10% of cases with LGA or small-for-gestational age (SGA) newborns, and that therefore a weight gain lower than the IOM recommendations in these patients could not be associated with negative effects on fetal growth and risk of cesarean section (74). Hinkle et al. showed how weight loss correlates with a higher incidence of SGA newborns in women with grade I obesity, for whom the acceptable GWG was between 0.1 and 4.9 kg. In contrast, in the class 2 and 3 obese (BMI> 35 kg/m^2^) a weight loss greater than 5 kg correlated with higher risks of SGA. These results suggested that the acceptable weight change in classes 2 and 3 obesity should be between −4.9 kg and + 4.9 kg (106).

A meta-analysis including over 1 million obese women confirmed that a GWG lower than the recommendations or a weight loss were associated with an increased incidence of SGA (OR 1.27 and 1.79 respectively), but also with a significant reduction in LGA, macrosomia and cesarean delivery (107). More recently, a French study on 996 pregnant women with class 2 obesity confirmed that gaining less than IOM guidelines (0–5 Kg) might reduce the risk of macrosomia, without increasing the risk of SGA, but with no other beneficial effect on maternal-neonatal outcomes at term. Only a weight loss during pregnancy correlated with a significant risk of infants <10^th^ percentile in class 2 obese (108).

Therefore, we suppose that less than recommended weight gain or weight loss in class 3 obese women might be acceptable in pregnancy if individualized, associated with the prevention of ketosis through adequate carbohydrates intake and monitoring of regular fetal growth. A moderate caloric restriction (20–25 Kcal/Kg) ensuring an adequate protein and carbohydrate intake for gestational age, may represent the right strategy to manage weight gain in pregnancy. Even in obese pregnant women, the caloric and carbohydrates intake must not fall below 1,600 kcal/day and 175 g/day respectively, with an adequate micro- and macronutrients intake, to prevent the risk of malnutrition, ketosis, and SGA infants (109).

### Nutritional Requirements for Obese Pregnant Women

Neural tube defects (NTD) is a well-known consequence of folate deficiency, and obese women have about a two-fold higher risk to deliver an infant with NTDs (110), because of an impaired body distribution of folate (111, 112), and a reduced placental transporter activity of folate (113). Several studies showed a hypofolatemia and a higher mean erythrocyte folate concentration (the latter expression of long-term consumption and tissue stores) among obese women in childbearing age (112) and in early pregnancy (114). There is no agreement among the guidelines on the intake of folic acid in pregnancy complicated by obesity (5 mg vs 0.4 mg) (115). Given that organogenesis is mostly completed within the 9th week of pregnancy, it is even more important that obese women begin folic acid supplementation at least 3 months before conception until the 12^th^ week (116).

Maternal obesity also negatively correlated with plasma vitamin B12, that was more likely to be deficient in women with a BMI ≥ 30.0 kg/m^2^ as compared to normal-weight. Low plasma levels of vitamin B12 have been associated with a 2.5–3 times greater risk of NTDs, independently of low folate levels, maybe contributing to the increased incidence of NTDs in obese women (117), and suggesting that obese women may need peri-conceptional supplementation of vitamin B12.

Obese pregnant women were more likely to have inadequate levels of 25(OH)D vitamin in pregnancy (118). Previous studies reported a decreased bioavailability of vitamin D from cutaneous stores or deposition in body fat (119). Bodnar et al. demonstrated that there was a twofold increase in maternal and neonatal vitamin D deficiency as maternal BMI increases from 22 to 34 kg/m^2^ (120). Vitamin D deficiency worsens obstetric outcomes: the incidence of cesarean section grows for intrapartum complications and increases the risk of preeclampsia (121). Although even in this case there is no univocity in the nutritional recommendations for obese pregnant women, prophylaxis with vitamin D should be provided from the beginning and for the entire duration of pregnancy, especially in women with BMI ≥30 kg/m^2^.

In summary, in obesity-complicated pregnancy, the weight targets should be individualized and planned from the beginning of pregnancy. Therapeutic interventions based on a dietary approach, physical activity or both, are able to reduce the risk of excessive weight gain, with a consequent reduced incidence of pregnancy complications. According to the ACOG guidelines, all obese pregnant women should receive early nutritional counselling and guidance on physical activity (115). However, most maternal and neonatal complications correlate with maternal BMI; therefore dietary-behavioural intervention is an essential strategy in obese women, but it should begin in the pre-conception period. A targeted approach to pre-conception weight loss reduces the risk of GDM, hypertension and macrosomia, therefore of obesity-related congenital abnormalities, such as neural tube defects, cardiac anomalies, anorectal atresia, and limb defects (86).

## Nutrition in Pregnancy Complicated by Gestational Diabetes

The diet of pregnant women does not differ from general population, even if it must meet the needs of fetal growth and ensure the course of the pregnancy itself. If pregnancy is complicated by hyperglycemia, the diet must also ensure adequate glycemic targets and therefore represents the cornerstone for the management of GDM.

### Optimal Diet for GDM Women

There is no clear evidence on the most appropriate nutritional approach to reduce the risks of negative maternal-neonatal outcomes, such as preeclampsia, macrosomia, and perinatal complications.

A meta-analysis by Yamamoto and collaborators (122) defined the Medical Nutritional Therapy (MNT) for GDM as the effective intervention to achieve maternal euglycemia and optimal neonatal weight, through an early intervention that also affects fetal programming. Although the efficacy of the dietary intervention is confirmed in the GDM, International Guidelines reported an extreme heterogeneity among nutritional indications: they were often contradictory and did not constitute solid evidence; in any case, they were mainly based on the control of the carbohydrates amount, and the role of others macronutrients and food quality were not evaluated (123). In addition, there is extreme heterogeneity in the recommended intake of carbohydrates (from 26 to 60% of daily calories), without considering the need for low glycemic index (LGI) choices (**Table 2**).

**Table 2:** Nutritional Medical Therapy in GDM: summary of the studied dietary approaches and related effects in pregnancy.

Diet	Composition	Benefits	Negative effects
**LCD**	Carbohydrates <35–45% of daily calories in pregnancy	- ↓ Post-prandial glycemia - ↓ LGA risk	- No safety evidence - Excessive nutrients restriction - Cause of anxiety in the pregnant woman and consequent reduced compliance - Lack of fibers - ↑ dietary fats - No evidence on long-term adherence
**LGID**	Free carbohydrates intake, rather foods with GI < 55	- Better glycemic control - ↓ Post-prandial glycemia, ↑ insulin sensitivity, ↓ insulin therapy needs. - Non-restrictive and associated with better compliance - Rich in fibers	Clear evidence about safety
**Caloric restriction**	Severe caloric restriction: <1500 kcal/die or 50% reduction of pre-gravidic caloric intake. Mild caloric restriction: 1600-1800 kcal/die or 30% caloric reduction	- Better glycemic control in obese women	- No clear additional benefits in maternal and neonatal outcomes - Risk of ketosis - To balance with adequate weight gain
**Mediterrean diet**	High consumption of vegetables, fruit, dried fruit, legumes, extra virgin olive oil and whole grains. Moderate intake of animal proteins and dairy products. Limitation of sugars.	- Better cardiovascular safety profile - High safety profile in pregnancy	Clear evidence about safety Lack of randomized trials in pregnancy

Adapted from Mahajan et al. (123). LCD, Low Carb Diet; LGID, Low Glycaemic Index Diet.

A recent review compared the different nutritional approaches in GDM, on both daily glycemic control and neonatal outcomes (123). The recommendations on carbohydrate restriction (Low carb diet- LCD <35–45% of daily calories) had poor evidence on efficacy and safety, and were associated with poor patient adherence and risk of replacement of carbohydrates with fats, which are metabolically harmful. The low glycaemic index diet (LGID), with at least 50% of daily calories of complex carbohydrates and high fiber intake, has been shown the most effective factor on the daily glycemic control of GDM women, and on the improvement of insulin sensitivity. It was also associated with better adherence because of the less restrictive carbohydrates intake. These conclusions confirmed previous reports showing less frequent insulin use and lower birth weight in GDM women following a LGID (124).

A pilot study by Hernandez and colleagues (125) randomized obese GDM women to a LGID rich in carbohydrates, versus a “traditional” diet with <40% carbohydrate and high fat content. After 7 weeks, pregnant women randomized to LGID had lower fasting and post-prandial blood sugar levels, lower insulin resistance, and less need for insulin therapy.

This evidence confirms that LGID, when combined with higher quality carbohydrates, lower fat, appropriate caloric intake, and ethnically suitable foods, allows to reach better metabolic targets, without micro- and macronutrient deficiency and risk of ketosis, particularly in GDM pregnant women because of increased insulin resistance (126).

### GWG in Women with GDM

No specific weight targets have been defined for GDM women, besides those related to pre-pregnancy BMI (IOM 2009) (76). Recent evidence suggests that stricter control of GWG could lead to a reduction in the incidence of negative neonatal outcomes in women with GDM, particularly if associated to obesity. A Chinese study found that GWG targets of 1 and 2 kg below the upper and lower limits of the IOM recommendations were associated with decreased adverse outcomes (LGA, macrosomia) (127).

Several studies support the benefit of more stringent GWG recommendations. Kurtzhals et al. investigated the impact of maternal GWG during dietary treatment on fetal growth, in pregnancies complicated by GDM. They found that restricted weekly GWG during dietary treatment was associated with decreasing HbA1c (p = 0.001) from diagnosis of GDM to late pregnancy and infants with a lower birthweight-SD score (0.15 ± 1.1), without increased prevalence of SGA infants (128).

However, controversies still exist, because an improvement in perinatal outcomes has not always been shown by assuming more stringent weight targets in GDM women (129). GWG and GDM are independent predictors of adverse pregnancy outcomes (particularly LGA newborns), but several other factors may have a role, including maternal glycemic control, pre-pregnancy BMI and maternal hypertriglyceridemia (36). Probably, more restrictive GWG targets may be considered in GDM, especially among obese pregnant women, to achieve a better metabolic control and minimise postpartum weight retention, hence long-term maternal morbidity related to obesity and T2D.

### Nutritional Requirements and Role of Nutraceuticals in GDM

During pregnancy, the micronutrients requirements increase even more than the total energy needs. Some nutraceuticals have been of particular interest to a possible role in the prevention or treatment of GDM.

Several studies explored the relationship between Vitamin D and GDM, hypothesizing that early supplementation can prevent the onset of the disease in women at risk. Higher levels of Vitamin D at early pregnancy seem to be associated with a lower risk of cesarean section, preterm birth, low birth weight (130), and with a lower risk of preeclampsia (131). There were major concerns about the role of Vitamin D in the risk of developing diabetes in pregnancy: two recent reviews and meta-analyses concluded that maternal Vitamin D deficiency was closely associated with high risk of GDM (132, 133). Particularly, Sadeghian et al. observed a 2% lower risk of GDM per 10 nmol/L increment of circulating 25(OH)D (132).

However, the optimal 25(OH)D levels for GDM prevention remain unknown. A European multicenter RCT of early intervention (before 26^th^ week of pregnancy) randomized women at risk for GDM (BMI ≥ 29 (kg/m^2^) with various intervention arms, including diet+physical activity+vitamin D and vitamin D alone (DALI study) (134). Unfortunately, supplementation with 1,600 IU/day of vitamin D did not show a clear effect in reducing the risk of GDM.

In another Chinese prospective birth cohort study on 4984 pregnant women, it was shown that GDM risk was significantly reduced only in pregnant women with 25(OH)D concentrations >50 nmol/L and taking 400–600 IU vitamin D/d, compared to women with mean 25(OH)D concentration <40 nmol/L (135).

Thus, there is some evidence from observational studies about the associations between vitamin D serum levels and maternal outcomes, such as GDM development; however, an overview of several systematic reviews, examining effectiveness from RCTs on 25(OH)D supplementation, showed no effect in pregnancy (136).

As far as the role of Vitamin D in GDM treatment is concerned, the results are even less conclusive. Several studies didn’t find a clear benefit on glycemic control with 25(OH)D supplementation in GDM women (137).

A meta-analysis encompassing RCT with planned 25(OH)D supplementation alone, concluded that Vitamin D treatment was associated with a decrease in fasting blood glucose by 0.46 mmol/L (−0.68, −0.25) (p < 0.001), glycated haemoglobin by 0.37% (−0.65, −0.08) (p < 0.01) and serum insulin by 4.10 µIU/mL (−5.50, −2.71) (p < 0.001) as compared to controls. However, the conclusion should be assumed with caution due to the limited number of included studies (138).

Greater efficacy would result from the co-administration of 25(OH)D and other micronutrients. Vitamin D and omega-3 fatty acids co-supplementation for 6 weeks in GDM patients demonstrated beneficial effects on fasting plasma glucose, serum insulin levels, HOMA index, serum triglycerides, and VLDL levels (139). Other studies about calcium+Vitamin D supplementation in women with GDM found beneficial effects on metabolic profile, such as reduction in fasting plasma glucose, plasma insulin, HOMA index, and LDL cholesterol (140), or lower rate of cesarean section, macrosomia, hyperbilirubinemia, and hospitalization in newborns (141).

Further studies are needed to fully understand the exact mechanism by which Vitamin D influences glucose metabolism and the optimal supplementation for GDM prevention.

Supplementation of polyunsaturated fatty acids (PUFAs) in GDM-complicated pregnancies seems to have some benefits on neonatal outcomes, although future studies on the effects of omega-3 in metabolic complications of pregnancy will be needed.

GDM is commonly associated with alterations in the lipid profile, with significantly increased levels of triglycerides and LDL cholesterol compared to women with normal glucose tolerance, and associated with higher values of adipocyte fatty acid-binding protein (AFABP) and other adipokines (142).

Studies on the association between the intake of DHA-PUFA and the risk of GDM have yielded conflicting results. Observational studies showed that a reduced dietary intake of PUFA, associated with an increase in saturated fats intake, represented a risk factor for the development of GDM (143). Other prospective studies have not identified this association (144).

Randomized trials did not conclude that PUFAs are effective in reducing the risk of impaired glucose metabolism. Zhou et al. randomized 2,399 women to take either 800 mg/day of DHA or DHA-free vegetable oil capsules, and concluded that the risk of GDM cannot be reduced by PUFA supplementation in the second half of pregnancy (RR 0.97) (145). Another Iranian study on the effects of omega-3 (1,000 mg, including 120 mg and 180 mg of DHA and EPA, respectively, for 6 weeks) on glucose metabolism in 56 women with GDM, did not show the efficacy of supplementation on the reduction of fasting insulin, insulin sensitivity or lipid profile, but confirmed the efficacy in reducing inflammation parameters in the intervention group (hs-PCR −236.3 ± 1,541.9 vs. 898.6 ± 2,292.7 ng/mL, P = 0.03) (146).

Either the heterogeneity of the study designs, in terms of the composition of supplementation (dosages and associations of DHA and EPA), or the limited number of the enrolled sample or the different gestational ages, could be associated with the lack of conclusive results on the efficacy of PUFA supplementation in GDM.

A promising supplement for GDM prevention and treatment is Inositol, a compound of the Vitamins B family, produced in large quantities in the body starting from D-glucose. In the body, 99% of inositol is present in the form of the myo-inositol isomer (MI), the remaining 1% is in the form of D-chiro-inositol (DCI). There are growing evidences that MI, combined with an appropriate dietetic regimen for GDM, can provide additional benefits.

A 2015 Cochrane Review included four prospective randomized trials (RCTs), conducted in Italy and involving 502 women, and showed that inositol supplementation in early pregnancy reduced the incidence of GDM in patients at risk (RR 0.43) (147). At the OGTT, a reduction in fasting (−4 mg/dl), at 1h (−12 mg/dl) and at 2h (−14 mg/dl) blood glucose was observed in the treated patients. There were no differences in terms of maternal-fetal outcomes. A more recent meta-analysis (5 RCTs, 956 participants) confirmed the safety of MI, proposing it as a prevention strategy, as it was associated with a reduction in the GDM incidence (OR 0.49) (148). A comparison among the different inositol stereoisomers (MI, DCI, combined MI+DCI or placebo) for GDM prevention demonstrated the largest benefit in the MI supplementation, with a lower incidence of abnormal OGTTs (149).

The role of MI as a non-pharmacological treatment for GDM is more controversial. A 2016 systematic review (150), including only 2 studies on MI as treatment of GDM *vs* placebo, did not identified differences between treated women and controls, in terms of GWG, need for insulin therapy, birth weight, LGA, preterm labor. There was also limited evidence regarding the effects of MI on fasting glucose, BMI, and neonatal hypoglycemias.

A recent Italian study evaluated the effects of the intake of 500 mg of DCI twice a day on metabolic control and maternal-fetal outcomes in 137 women with GDM, showing better glycemic control in pregnancy and a better trend in maternal weight and fetal growth compared to placebo (151).

Eventually, there is a lack of trials investigating the effect of interventions prior to or between pregnancies on risk of GDM (152). Further studies will be needed to examine the effectiveness of different nutraceuticals supplementation in prevention and treatment of GDM.

## Ante-Natal Nutrition, Obesity, and GDM: Long-Term Effects on Offspring

The DOHaD hypothesis conceptualized the intra-uterine basis of long-term metabolic programming in progeny: it has become increasingly evident how this developmental plasticity allows the fetus to arrange anticipatory responses to the external environment, already altering the cell differentiation pathway and acquiring adaptive advantages to the challenges of adult life. Any discrepancy between the pre-natal and post-natal adaptation environment will cause disease.

The concept of “fetal programming” stems from the assumption that fetal development is composed of some critical periods during which every system or organ is formed. These periods are short and occur at different times for each system, although most of these take place *in utero*. After birth only brain, liver and immune system remain plastic, therefore there are 3 reasons why low-weight infants will be more vulnerable in adulthood: they have a reduced functionality of some organs (e.g. kidney), show alterations in hormonal feedback and metabolic structure, and remain more vulnerable to adverse environmental influences in later life. The child development has a sort of hierarchy of priorities: brain growth is at the top of this hierarchy, and the development of organs like kidney and lungs, which don’t work in the womb, are at the bottom. By triggering adaptation mechanisms to the environment, such as either a decrease in vascular bed, or a reduction in the number of nephrons, or changes in insulin secretion, the fetus is able to conserve the available energies for neuronal development and cardiac function (13).

The DOHaD theory has gained attention after the first major study on the effect of maternal-fetal malnutrition on metabolic outcomes in adult life, the “Dutch famine birth cohort Study”, in which, in Amsterdam, 2000 newborns during the Second World War (November 1943-February 1945) were evaluated and followed until 1996. Under the Nazi embargo, maternal nutritional intake did not exceed 400-800 kcal/day: individuals subjected to calorie restriction during intrauterine life showed an increased risk of developing cardio-vascular diseases, but also other complex pathologies, such as schizophrenia and early cognitive impairment (153, 154). The consequences were different according to the different gestational ages affected by nutritional deficiency. Babies whose mothers were malnourished in the middle or late part of the pregnancy, had normal birth weight and developed impaired glucose tolerance in adulthood. On the other hand, subjects whose mothers had been a caloric restriction at early stage of pregnancy showed an increased BMI and a more atherogenic lipid profile.

The possibility that early exposure confers a long-term risk of complex diseases, such as obesity, diabetes and cardiovascular diseases, implies that the changes in metabolic processes and neuroendocrine functions, which underlie this susceptibility, are persistent. Epigenetic mechanisms (DNA methylation, changes in histone proteins, and non-coding RNA or miRNA expression), causing persistent changes in the regulation of genetic pathways, can be transmitted during cell division and are probably also inheritable throughout generations, therefore justifying the associations between adult diseases and adverse nutritional or environmental conditions during intra-uterine development. Heijmans and colleagues reported how the individuals of Dutch famine follow up cohort study, after sixty years, had significantly reduced methylation of the imprinted gene for Insulin-like Growth Factor II (IGF2, locus IGFII-H19) compared to subjects who had not undergone the same deprivation in womb (155). IGF2 is a determining factor in human growth and development, and has a maternal imprinting, playing a key role in cardio-vascular diseases.

### Obesity, Overnutrition, and Fetal Programming

It is well known that both under- that over-nutrition have detrimental effects on metabolic health and on the risk of offspring obesity. Maternal obesity affects fetal metabolism and can cause changes in the amount of nutrients and metabolites, passing through the placenta.

A meta-analysis of data from 162,129 mothers and their children from 37 pregnancy-birth cohort studies in Europe, North America, and Australia showed that higher maternal pre-pregnancy BMI and GWG were associated with the increased risk of childhood overweight/obesity, with strongest effect on later ages (10–18 years) (156).

Developmental programming due to maternal obesity probably occurs throughout gestation, defining the crucial periods during which the major changes initiate, that are keys to plan effective interventions. Obesity in pregnancy is characterised by dyslipidemia, hyperleptinemia, hyperinsulinemia and a proinflammatory state, that may enhance adipogenesis and lipogenesis, resulting in higher white adipose tissue mass and adipocyte hypertrophy in offspring (157). Higher leptin levels were found in macrosomic newborns and are related to insulin levels and adiposity (158); this may explain how fetuses of obese mothers develop insulin-resistance in the womb and are born with higher adiposity (159).

In fact, not only in the context of obesity, the maternal diet can affect fetal metabolism, in terms of both an increased supply of nutrients and an altered expression of fetal genes through epigenetic mechanisms. Nutritional, environmental and epigenetic factors interact with each other and may cause to induce obese phenotypes because of epigenetic modifications *in utero*, with transgenerational effects.

Maternal overnutrition resulted in an increase in the fetal expression of the adipogenic transcriptional factor, PPARγ, and in lipoprotein lipase, adiponectin and leptin in adipose tissue and these changes may lead to obesity in later life (160, 161).

Animal models allow us to understand many of these mechanisms. In the rodent model, offspring from mothers fed with a high-fat-diet (HFD) before and during gestation show a rapid weight gain during lactation, according to a key period of adipose tissue development. Moreover, adult male offspring from HFD mothers are predisposed to fat accumulation, with an increased visceral, gonadal and perirenal fat depots, and with hyperleptinemia: the adipose tissue depots exhibits elevated sterol regulatory element-binding protein 1 (SREBP1), fatty acid synthase (FAS), leptin, and diminished PPARγ mRNA levels (157), establishing the basis for metabolic diseases in adulthood.

Actually, the earlier the excessive fat intake in the maternal diet begins, the greater the long-term damage. The fetal liver is uniquely vulnerable to dysregulated fuel metabolism caused by excess fuel exposure due to maternal obesity or high-fat diet: it has fewer mitochondria, immature antioxidant defence system, little or no gluconeogenesis and limited lipid oxidation; moreover, fetal adipose depots in the first half of pregnancy are immature and not available to buffer the excess transplacental lipids or other fuels in maternal obesity until late gestation. Thus, early increased lipids, due to maternal obesity or HFD, probably accumulate in the fetal liver, establishing an inflammatory and lipotoxic environment and priming Kupffer cell and hepatic stellate cell to fibrosis, inflammation and non-alcoholic fatty liver diseases (NAFLD) later in life (162).

Maternal HFD diet may impact the hypothalamic control system of energy balance in the offspring too. It was found that early excessive energy intake, along with maternal obesity, is associated with changes in hypothalamic regulation of body weight and energy homeostasis, by altering the expression of leptin receptor, proopiomelanocortin (POMC) genes and neuropeptide Y in offspring by causing epigenetic changes in DNA methylation (163, 164). In this way, gestational HFD is likely to cause hyperphagia, increase in FM, and insulin resistance in offspring.

### GDM and Metabolic Effects on Offspring

There is growing interest in understanding the long-term effects of hyperglycemia and GDM in pregnancy: however, there is difficulty in limiting the confounding factors related to obesity and maternal overnutrition, frequently associated as unique manifestations.

Epidemiological studies showed that offspring of GDM pregnancies had significantly higher prevalence of Metabolic Syndrome at around age 11 (165) particularly when associated to maternal obesity (166). A study comparing metabolic outcome in offspring 15 years after GDM pregnancies observed that daughters of mothers with GDM had higher waist circumference and insulin resistance compared to daughters of non-GDM mothers, but maternal BMI was the strongest predictor of child adiposity (167). An association between maternal GDM and overweight status in the offspring has been observed in other cohorts (168, 169).

Interestingly, follow-up data from the HAPO study on over 4000 adolescents showed that 10.6% of children born to mothers with untreated GDM had impaired glucose tolerance (IGT) compared to 5.0% of children of mothers without GDM, and they were more insulin resistant with limited β-cell compensation (170). Moreover, exposure to higher levels of glucose *in utero* was independently associated with adiposity at 10–14 years of age, (obesity, skinfold thickness, per cent body fat and waist circumference) also after adjustment for maternal BMI (171). Glucose levels also less than those diagnostic of GDM resulted associated with greater childhood adiposity, with obvious implications for long-term metabolic health.

A more recent sub-analysis of HAPO FU on 4,832 mothers with untreated GDM and their 10–14 years old children, confirmed that BMI and glycemia, measured mid-pregnancy, were joint predictors of childhood adiposity outcomes at mean age 11.4 years (172). The original HAPO Study demonstrated that the combination of untreated GDM and maternal obesity was associated with higher odds for birth weight, newborn body fat, and cord C-peptide >90th percentile, compared with the odds for either GDM or obesity alone, suggesting a more than additive association of GDM and obesity with neonatal outcomes (90). The analysis of Josefson et al. demonstrated that additive associations of maternal obesity and untreated GDM with adiposity outcomes persist into childhood (172).

Also for GDM, epigenetic mechanisms have been involved by several studies. For example, the leptin gene (LEP) seems to have different methylation levels in women with GDM: maternal glucose values correlate positively with the methylation of the LEP gene in placental tissues and negatively in fetal tissues (173). Methylation of the promoter of the gene for adiponectin (ADIPOQ) is reduced in the fetal placenta of mothers with hyperglycemia, while hypomethylation of the same gene on the maternal side of the placenta correlates with the HOMA index and insulin resistance (174).

The methylation levels of the ABCA1 gene, a cholesterol efflux regulator involved in the pathogenesis of atherosclerosis, negatively correlates with maternal HDL and positively with glycemic levels. On the fetal side, placental ABCA1 methylation negatively correlates with cord blood triglyceride levels, while methylation of the same gene in cord blood cells negatively correlates with maternal blood glucose (175).

Genome-wide methylation studies revealed an increased global DNA methylation in placenta of GDM mothers (176) and significant methylation differences in fetal cord bloods (177), while in the offspring several functional networks, which are epigenetically programmed through GDM exposure, were identified (178, 179).

## Conclusion

The first 1,000 days of life (from conception up to two years of life) are essential for the prevention of disease in adulthood. We should be aware that specific maternal conditions during the pre- and peri-conceptional period (particularly obesity and excessive weight gain during pregnancy) are associated with higher risk of maternal complications and LGA infants, with obesity and impaired glucose metabolism in children and, subsequently, with increased cardiometabolic risk in adults.

Understanding the way leading from derangement in physiological mechanisms of metabolic and endocrine adaptation to the metabolic complications of pregnancy, becomes the focal point for all the strategies that we must early implement, virtually before conception, to safeguard the health of both mother and progeny.

In the era of exponential development of DOHaD theory, where understanding epigenetic and gene expression mechanisms is spreading in scientific discussion, a new frontier in research is about the paternal role in long-term metabolic risk transmission (180): the new concept of PHOaD—Paternal origin of health and diseases (181). This highlights the need for an early nutritional management intervention, which theoretically includes the couple from pre-conceptional stages, and which subsequently involves the mother from conception to birth, to ensure the prevention and treatment of any metabolic imbalances.

This fascinating research lead us to embrace the perspective of biological parenting beginning long before birth, even before conception, and to define prevention protocols that make it possible to reduce the impact of metabolic and cardiovascular diseases.

## Author Contributions

SP and ET conceived the study. AC did the literature search. SP and ET wrote the manuscript. All authors contributed to the article and approved the submitted version.

## Conflict of Interest

The authors declare that the research was conducted in the absence of any commercial or financial relationships that could be construed as a potential conflict of interest.
